# Kappa Distributions and Isotropic Turbulence

**DOI:** 10.3390/e21111093

**Published:** 2019-11-07

**Authors:** Elias Gravanis, Evangelos Akylas, Constantinos Panagiotou, George Livadiotis

**Affiliations:** 1 Department of Civil Engineering and Geomatics, Cyprus University of Technology, PO Box 50329, Limassol 3603, Cyprus; evangelos.akylas@cut.ac.cy; 2 Nireas International Water Research Center, Department of Civil and Environmental Engineering, University of Cyprus, 75 Callipoleos, Nicosia 1678, Cyprus; 3 Division of Space Science and Engineering, Southwest Research Institute, San Antonio, TX 78238, USA; glivadiotis@swri.edu

**Keywords:** isotropic turbulence, structure functions, PDF, DNS, Karman–Howarth equation, kappa distribution, kappa index, superstatistics

## Abstract

In this work, the two-point probability density function (PDF) for the velocity field of isotropic turbulence is modeled using the kappa distribution and the concept of superstatistics. The PDF consists of a symmetric and an anti-symmetric part, whose symmetry properties follow from the reflection symmetry of isotropic turbulence, and the associated non-trivial conditions are established. The symmetric part is modeled by the kappa distribution. The anti-symmetric part, constructed in the context of superstatistics, is a novel function whose simplest form (called “the minimal model”) is solely dictated by the symmetry conditions. We obtain that the ensemble of eddies of size up to a given length *r* has a temperature parameter given by the second order structure function and a kappa-index related to the second and the third order structure functions. The latter relationship depends on the inverse temperature parameter (gamma) distribution of the superstatistics and it is not specific to the minimal model. Comparison with data from direct numerical simulations (DNS) of turbulence shows that our model is applicable within the dissipation subrange of scales. Also, the derived PDF of the velocity gradient shows excellent agreement with the DNS in six orders of magnitude. Future developments, in the context of superstatistics, are also discussed.

## 1. Introduction

Isotropic turbulence is an idealized form of turbulence which is defined by the invariance of the scalar correlation functions of the velocity field under translation, rotation, and reflection [1,2]. It is the simplest form of turbulence. It can be approximately realized when the mean flow is zero or uniform, and occurs at scales much smaller than the scale where the stirring forces are applied. The most faithful realization of the isotropic turbulence is represented by the direct numerical simulations (DNS) of turbulence [3]. In the DNS, the Navier–Stokes equations are solved in a cubic box with periodic boundary conditions. Energy is fed in the flow at scales of the size of the box. In the recent years, high resolution DNS for (Taylor scale) Reynolds numbers larger than 1000 have appeared, see e.g., [4,5,6,7,8]. Those DNS allow for clarification of phenomena of isotropic turbulence, such as the bottleneck effect, see e.g., [9], as well as better determination of important characteristics of turbulence, such as the probability density function (PDF) of the distribution of the velocity derivative, including its skewness and flatness factors, as well as of the PDF of the one-point velocity distribution [5]. The one-point velocity PDF is nearly Gaussian, with a flatness factor somewhat below 3, depending on the Reynolds number, while the velocity derivative PDF is well known to deviate significantly from the Gaussian, see e.g., [10].

Although extensive theoretical work has been devoted to determine the one-point PDF of the velocity field (that is, the PDF of the value of the velocity field at one point), see e.g., [10], simple direct modeling is rather sparse [11,12,13]. In the present work we attempt to describe the two- and one-point PDFs (that is, the PDFs of the value(s) of the velocity field at one and two points, respectively), based on the theory of the kappa distribution which has been very useful in describing space plasmas, see e.g., [14,15] and the references therein. The kappa distribution has a parameter additional to temperature, called the kappa index, and coincides with the Maxwell–Boltzmann (MB) distribution for the infinite kappa index. The thermodynamic origin of the kappa distribution, and the nature of the kappa index as a second intensive parameter (thermodynamic integral) was clarified in [16] allowing for pseudo-additivity of the entropy function. A very convenient interpretation of the kappa distribution follows from the concept of ‘superstatistics’: if the temperature of am MB system is fluctuating in a random way according to a prescribed distribution, then the system is described by kappa distribution if the inverse temperature follows a gamma distribution [15,17]. The physical interpretation of this construction clearly involves an ensemble of systems which are not in classical thermodynamic equilibrium. From a pragmatic point of view, the kappa distribution is a family of functions labeled by the kappa index which exhibits fatter tails than an MB distribution, and can be understood as a superposition of MB distributions.

In the present work, we attempt to model the two-point joint probability distribution of the velocity field in isotropic turbulent flows using kappa distribution and superstatistics as building blocks. The two points refer to geometric points in physical space, that is, we attempt to model the PDF for two velocity variables as a function of the distance between the observation points, given that isotropic turbulence is also homogeneous. In particular, the aim is to associate the parameters of the kappa distribution i.e., the temperature parameter and the kappa index, with fundamental quantities (functions) of isotropic turbulence. One must emphasize that in the context of the incompressible flows we are considering here, the temperature parameter does not correspond to an actual thermodynamic temperature, but rather a quantity of the proposed statistical description with formal properties quite analogous to the thermodynamic temperature, as emerges in statistical mechanics. Hence, the temperature parameter may also be referred to as statistical temperature, which is associated with the mean square of the velocity of a unit mass volume of the fluid, and it is a measure of the intensity of turbulence. On the other hand, the actual thermodynamic temperature is associated with the mean square of the velocity of the molecules of the fluid. That is, the temperature parameter refers to entirely different scales of motion from the thermodynamic temperature, i.e., the scales of the eddies of the turbulent flow.

The work of Kolmogorov [1,2,18] provided the theoretical framework within which one usually thinks about isotropic turbulence (known in the literature of turbulence as K41). The Kolmogorov laws are expressed in terms of the structure functions, that is, statistical averages of integral powers *n* of the velocity increment between two points, or in terms of the Fourier transform of those functions. The number *n* is called the order of the structure function. As mentioned, due to isotropy and homogeneity, quantities such as the structure functions depend only on the distance between the two points. Kolmogorov’s hypotheses [1,2,18] imply that, in a certain intermediate range of scales, called the inertial subrange, in which energy primarily cascades to the smaller scales with negligible viscous dissipation, the structure functions obey simple scaling laws. Those laws have been verified by numerical and actual experiments [6,10], although discrepancies from K41 have been found, which have been attributed to the intermittent character of turbulence in space and time (for a discussion on proposed theories see e.g., [2]). In the small scales (which are quantified by the Kolmogorov dissipation scale), where the dissipation of energy is dominant, the Navier–Stokes equations imply that the structure functions are analytic (odd or even) functions of the distance. Certain coefficients in their expansion in power series of the distance are related to fundamental scalar quantities of isotropic turbulence, such as the skewness and the flatness factor of the velocity gradient distribution, which are clearly associated with the smallest distances. In what follows, only the general properties of the structure functions, stemming from the statistical theory of the Navier–Stokes equations, will be important, while the details of the K41 theory will mainly serve as source for certain additional comments in order to put the proposed theory in a specific context. 

One may then proceed to investigate the possibility of describing isotropic turbulence with the kappa distribution and superstatistics. Reflection symmetry implies that the PDF for the values of the velocity field at two points must be invariant under an exchange of the velocity variables and a simultaneous change of their sign. This implies that the two-point PDF must be the sum of a symmetric and even part, denoted ‘*se*’, and an antisymmetric and odd part, denoted ‘*ao*’. By ‘even’ we mean that function is unchanged by a change of sign of both velocity variables, while by ‘odd’ we mean a function that changes sign under a change of sign of both velocities. The even order *n* structure functions, which are even functions of the separation distances, are determined from the *se* part, while the odd order ones are determined by the *ao* part and they are odd functions. Hence, the theory is naturally divided into a symmetric and an antisymmetric sector, which may be modeled independently. 

The symmetric sector is easily modeled by the two-degree of freedom kappa distribution. We then learn that the temperature parameter of the kappa distribution equals the second order structure function *B*_2_(*r*), that is, it depends on the separation distance *r* between the two observation points. Hence, eddies of size up to *O*(*r*), may be thought of as an ensemble of statistical temperature *B*_2_(*r*). For large *r*, this temperature becomes 4/3 of the total energy per unit mass of flow. The fourth order structure function *B*_4_(*r*) turns out to be a function of both the temperature parameter and the kappa index. That implies that the ensemble of eddies of size up to *O*(*r*) have also an associated kappa index that depends on *r*. From the behavior of *B*_4_(*r*) and *B*_2_(*r*) for small distances, one obtains the kappa index of small eddies as a function of the flatness factor of the velocity gradient distribution, and hence of the Reynolds number utilizing modern DNS data [5]. 

The antisymmetric sector requires novel modeling, as the kappa distribution is symmetric. Guiding principles are the symmetry properties of the function ‘*ao*’ and a condition imposed on it following from the homogeneity of turbulence. Those restrictions are strong enough so that the simplest imaginable model (called the minimal model) that can be constructed in the context of superstatistics is effective enough. We then learn that: (i) The kappa index of the ensemble of eddies of size up to *O*(*r*) is naturally related to the third order structure function *B*_3_(*r*), through an important relation that involves also *B*_2_(*r*) and reminds one of the so-called ‘closure schemes’ of isotropic turbulence; (ii) this relation does not depend on the model, only on the superstatistics gamma distribution; (iii) the DNS data and self-consistency imply that the theory is valid for *r* up to ~10*η*, where *η* is the previously mentioned Kolmogorov dissipation range scale; (iv) the PDF for the velocity gradient can be derived from the two-point PDF in the limit *r*→0 (for the minimal model) which agrees well within six orders of magnitude with the DNS data for that quantity; (v) on the theoretical side, the Karman-Howarth equation relating the second and third order structure function of isotropic turbulence translates into a differential equation relating the temperature parameter and the kappa index functions. 

Finally, the one-point PDF for the value of the velocity field can be derived. It follows from the symmetric sector alone, and it is a kappa distribution of one degree of freedom with a temperature parameter and a kappa index that depends on the ensemble of eddies of size up to *O*(*r*) for any specific scale *r* of interest. Taking into account the whole of the flow, i.e., *r*→∞, we argue that the one-point PDF should be Gaussian. 

## 2. Structure Functions of Isotropic Turbulence

Consider an arbitrary axis in the turbulent flow, which we may call *x*, and the *x*-components of the velocity field at points *x_L_* and *x_R_* (the subscripts implying ‘left’ and ‘right’, respectively), denoted by *u_Lx_* and *u_Rx_*, respectively. The (longitudinal) structure functions of turbulence of order n are defined as

(1) $$B_{n}(r)=\langle {\left(u_{Rx}-u_{Lx}\right)}^{n}\rangle ,\;r=x_{R}-x_{L}$$

where *u_Rx_* = *u_x_*(*x_R_*) and *u_Lx_* = *u_x_*(*x_L_*). The brackets indicate an average over realizations of the turbulent velocity field. The quantity *r* is the distance between the two observation points. A few cornerstone results regarding structure functions can be summarized as follows.

The function *B*_2_ is an even function of *r*, and the lowest order term in a small *r* expansion is given by

(2) $$B_{2}=\frac{\varepsilon }{15\nu }r^{2}$$

where *ν* [L^2^T^–1^] is the kinematic viscosity of the fluid, and ε [L^2^T^–3^] is the energy dissipation rate per unit mass of the fluid. In the so-called inertial subrange the function *B*_2_ is given by the famous Kolmogorov result [1,2,3]

(3) $$B_{2}=C_{2}{\left(\varepsilon r\right)}^{2/3}$$

where *C*_2_ is the Kolmogorov constant, a pure number with a value around 2, that depends on the Reynolds number. The result in Equation (3) is derived essentially by scaling arguments. Important in our analysis, will be the Reynolds number of the Taylor micro-scale *λ*:

(4) Reλ=u′λν=15u′4εν=203K2εν,  λ=10νKε,  u′=〈ux2〉,  K=32u′2

The root mean square of the *u_x_* is independent of the location on the axis *x* by the homogeneity of the field. The quantity *K* is the kinetic energy per unit mass of the fluid. Due to the isotropy of the flow, the kinetic energy associated with each direction is the same. Finally, for *r*→∞ we have that *B*_2_ approaches a constant:

(5) $$B_{2}=2{u^{\prime }}^{2}=\frac{4}{3}K$$

The function *B*_3_ is an odd and always negative function of *r*, and the lowest order term in a small *r* expansion is of order r^3^ and it is best encoded in the following limit

(6) $$S=\underset{r\to 0}{\lim}\frac{B_{3}}{{\left(B_{2}\right)}^{3/2}}$$

which defines the skewness factor of the longitudinal derivative of the velocity field, ∂*u_x_*/∂*x*. The skewness *S* is a negative pure number, which depends on the Reynolds number and is around ½ for Taylor–Reynolds numbers in the range of hundreds. In the inertial subrange the function *B*_3_ is given by the Kolmogorov ⅘-law [1,2,3]

(7) $$B_{3}=-\frac{4}{5}\varepsilon r$$

Finally, the function *B*_4_ is an even function of *r*, whose small *r* behavior is best encoded via the ratio

(8) $$F=\underset{r\to 0}{\lim}\frac{B_{4}}{{\left(B_{2}\right)}^{2}}$$

which defines the flatness factor of the longitudinal derivative of velocity field, ∂*u_x_*/∂*x*. The flatness *F* is a positive pure number, which depends on the Reynolds number. The dependence of *S* and *F* on the Reynolds number is determined through direct numerical simulations of isotropic turbulence and is discussed below.

## 3. Two-Point Joint Probability Distribution for the Velocity Field: General Properties

The structure functions given by (1) are invariant under reflection, encoded by the transformation

(9) $$(u_{Rx}{)}^{\prime }=-u_{Lx},\;(u_{Lx}{)}^{\prime }=-u_{Rx}$$

**Figure 1:**
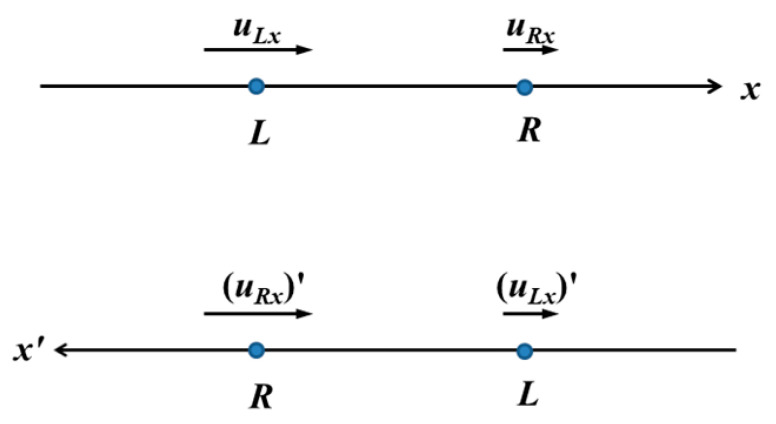
Transformation of velocities under reflection.

The transformation of the velocity variables under reflections is explained pictorially in Figure 1. As, we shall realize shortly, this symmetry is nearly trivial for the *n* = even order structure functions, but it is fundamental for the *n* = odd order structure functions. From here on we shall drop the index *x* for brevity.

Consider now a joint probability density function *p*(*u_R_*,*u_L_*) that encodes the statistics of the two velocity variables of the flow, so that

(10) $$B_{n}=\iint {\left(u_{R}-u_{L}\right)}^{n}p(u_{R},u_{L})du_{R}du_{L}$$

[Note: All integrations with respect to velocity variables refer to the interval (–∞, +∞)]. That means that the probability density must be invariant under reflections:

(11) $$p(u_{R},u_{L})=p(-u_{L},-u_{R})$$

Now, the question is: what are the properties of such a probability density?

To analyze that, we first need a few definitions. Consider general functions *p*(*u*,*v*) of two variables, and let us indicate by an index ‘*s*’, functions that are symmetric under an exchange of the two variables, and by an index ‘*a*’, functions that are anti-symmetric under an exchange of the two variables:

(12) $$p_{s}(u,v)=p_{s}(v,u),\;p_{a}(u,v)=-p_{a}(v,u)$$

Next, by an index ‘*e*’ we indicate functions which are even under a simultaneous change of sign of the two variables, and by an index ‘*o*’, functions which are odd under a simultaneous change of sign of the two variables:

(13) $$p_{e}(u,v)=p_{e}(-u,-v),\;p_{o}(u,v)=-p_{o}(-u,-v)$$

It is easy to see a general function *p*(*u*,*v*) of two variables can written as

(14) $$p(u,v)=p_{se}(u,v)+p_{so}(u,v)+p_{ae}(u,v)+p_{ao}(u,v)$$

where the index ‘*se*’ indicates a symmetric and even function according to the definitions above, the index ‘*so*’ indicates a symmetric and odd function, and so on.

Now, it is easy to see that only the *se* and *ao* components are invariant under reflections, that is, they identically respect Equation (9). Hence, we have that

(15) $$p(u_{R},u_{L})=p_{se}(u_{R},u_{L})+p_{ao}(u_{R},u_{L})$$

In all, invariance under reflections helped us specify the algebraic symmetries of the joint probability density function. We need now to clarify the implications of Equation (15).

**Remark** **1.** *By Equations (10) and (12), only the se component of the probability density specifies ‘n=even’ order structure functions, while only the ao component specifies the n=odd order ones:*

(16) $$B_{n=even}=\iint {\left(u_{R}-u_{L}\right)}^{n}p_{se}(u_{R},u_{L})du_{R}du_{L},\;B_{n=odd}=\iint {\left(u_{R}-u_{L}\right)}^{n}p_{ao}(u_{R},u_{L})du_{R}du_{L}$$

*This is due to symmetry properties of the factor (u_R_–u_L_)^n^ in each case, and can be verified by a suitable change of variables in the integrals, e.g., u_R_→u_L_ and u_L_→u_R_. Hence, we conclude that an ao component is necessary in order to have a non-zero function B_3_ and therefore a non-zero skewness S, which is a fundamental property of turbulence. A most important implication of (16) is that, in terms of modeling the probability distribution, we may think and model separately the symmetric sector associated with the se component and the ‘n=even’ order structure functions, and the anti-symmetric sector associated with the ao component and the ‘n=odd’ order structure functions.*

**Remark** **2.** *The ao component of the density function does not affect the normalization of the density function. Indeed,*

(17) $$\iint p_{ao}(u_{R},u_{L})du_{R}du_{L}=0$$

*as one may verify by a change of variables such as u_R_→u_L_ and u_L_→u_R_. Hence, one may only normalize the se component to the value 1.*

**Remark** **3.** *A non-trivial condition is imposed on the ao component by the fact that the one-variable probability distributions are the same for both L and R points, by the homogeneity of turbulence. That is, the functions*

(18) $$p_{R}(u)=\int p(u,v)dv,\;p_{L}(u)=\int p(v,u)dv$$

*must be the same: p_R_(u) = p_L_(u).*

*The se part of the probability density (15) is fully consistent with the condition p_R_(u) = p_L_(u). The ao part is not. Indeed, we first note that*

(19) $$\int p_{ao}(u,v)dv=\int p_{ao}(u,-v)dv=-\int p_{ao}(-u,v)dv$$

*where in the first equality we changed variables as v→–v and in the second equality we used the odd-ness of the ‘ao’ component. Equation (19) implies that the part of the function p_R_(u) that comes from the ao part of the joint probability density is an odd function. The same can be shown for the function p_L_(u). Secondly, we may note also that Equation (19) can be continued with a third equality*

(20) $$\int p_{ao}(u,v)dv=\int p_{ao}(u,-v)dv=-\int p_{ao}(-u,v)dv=+\int p_{ao}(v,-u)dv$$

*using the anti-symmetry of the function p_ao_ under an exchange of variables. That implies that*

(21) $$p_{R}(u)=p_{L}(-u)=-p_{L}(u)$$

*for the parts of the functions coming from the ‘ao’ component of the joint probability density. Therefore, those parts do not agree and must be zero. Hence, homogeneity imposes the following condition on the ‘ao’ component of the joint probability distribution:*

(22) $$\int p_{ao}(u,v)dv=0,\;\mathrm{for}\;\mathrm{all}\;u$$

*Integration with respect to the first variable is implied by (22) via the anti-symmetry of the function.*

**Remark** **4.** *Nothing in the previous conditions guaranties that the probability density (15) is everywhere non-negative. That needs to be imposed as a separate condition.*

## 4. Modeling the Symmetric Sector: *κ*-Distribution and Turbulence

The proposal is that the *se* component of the joint probability distribution for *u_R_* and *u_L_* is modeled by the two degrees of freedom kappa-distribution:

(23) $$p_{se}(u_{R},u_{L})=\frac{\kappa_{0}+1}{\pi \kappa_{0}\theta^{2}}{\left(1+\frac{u_{R}^{2}+u_{L}^{2}}{\kappa_{0}\theta^{2}}\right)}^{-\kappa_{0}-2}$$

As discussed in the introduction, the kappa distribution contains two parameters: the temperature parameter *θ*^2^ and the kappa index. From that one may draw a number of implications.

The second order structure function reads

(24) $$B_{2}=\iint {\left(u_{R}-u_{L}\right)}^{2}p_{se}(u_{R},u_{L})du_{R}du_{L}=\theta^{2}$$

That is, the temperature parameter *θ*^2^ equals *B*_2_, which in turn depends on the distance *r* and the energy dissipation rate *ε*. The function *B*_2_ contains information about eddies of size up to the distance scale *r*. On this basis one may state: The ensemble of eddies of size up to *O*(*r*) is an ensemble with statistical temperature *θ*^2^ = *B*_2_(*r*). (Note that the constant *κ*_0_ is not involved in this relation.) Ensembles of eddies of different sizes correspond to ensembles of different statistical temperatures. Turbulence is not in equilibrium from this point of view. As mentioned in the introduction, one must emphasize that in the context of the incompressible flows we are considering here, the temperature parameter does not correspond to an actual thermodynamic temperature, which is associated with the mean square of the velocity of the molecules of the fluid, but it is a statistical parameter, associated with the randomness of the motion of the eddies in the turbulent flow.

The fourth order structure functions reads

(25) $$B_{4}=\iint {\left(u_{R}-u_{L}\right)}^{4}p_{se}(u_{R},u_{L})du_{R}du_{L}=\frac{3\kappa_{0}}{\kappa_{0}-1}\theta^{4}$$

The relation between *B*_2_ and *B*_4_ as implied by (24) and (25) is too simple for hold for all *r* for constant *κ*_0_. Hence, we understand that *κ*_0_ depends on *r*, along with *θ*, although further information is needed to quantity that dependence:

(26) $$\kappa_{0}=\kappa_{0}(r)$$

where, as in the case of the temperature parameter *θ*^2^ = *B*_2_(*r*), we have omitted the obvious prior dependence on the Reynolds number, i.e., on the state of turbulence. We conclude that each ensemble of eddies of size up *O*(*r*) has its own temperature parameter and kappa-index. We shall see below that the kappa index *κ*_0_(*r*) is associated with the third order structure function *B*_3_(*r*).

Flatness can be then immediately calculated by Equations (8) and (25) from *r*→0:

(27) $$F=\frac{3\kappa_{0}(0)}{\kappa_{0}(0)-1}$$

Flatness is a function of *κ*_0_ alone. In the K41 isotropic turbulence, *F* can only be a function of the Reynolds number. Hence, *κ*_0_ is indeed a function of the Reynolds number as well. Experimental data and results from direct numerical simulations imply that [5,19,20]

(28) F=F(Reλ)~aReλα

for certain parameters *a* and *α* determined by best fit for a range of Reynolds numbers (up the order of 10^3^), although the most recent analyses [20] suggest that the flatness factor may asymptotically approach a constant value, in the spirit of K41. For illustration purposes, we may adopt the results of [5], as other types of data, such as the PDF of the velocity derivative, will also be taken from the DNS covered in [4,5,6,7]: *a* = 1.14 and *α* = 0.34. At the same time, one should bear in mind that the simple power law (28) is barely far beyond Reynolds number ~10^3^, thus it only indicates a trend.

**Figure 2:**
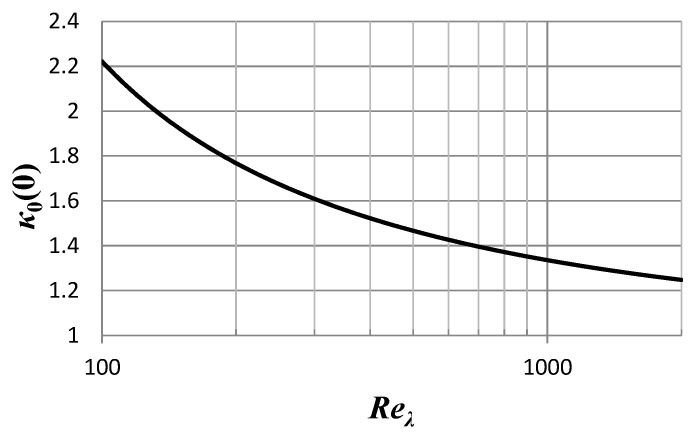
The dependence of the smallest scales kappa-index, *κ*_0_(0), on the Reynolds number, according to a power fit of the flatness factor, Equation (28), and according to the specific fit of [5], for a range of Reynolds the best fit of [5] that have been covered by the recent direct numerical simulations (DNS).

Hence, the small distances value of *κ*_0_ can be determined and in particular, given a specific dependence on the Reynolds number of the flow:

(29) κ0(0)=F(Reλ)F(Reλ)−3~1.14Reλ0.341.14Reλ0.34−3

emphasizing—as above—that the explicit estimate given in the last expression is only given for illustration purposes and it is not valid for Reynolds numbers ~10^4^ and larger. The magnitude and the trend of the dependence of the small distance *κ*_0_ on the Reynolds number is given in Figure 2: It apparently approaches asymptotically the value 1, although recent analysis [20] suggest that the flatness factor may reach a (finite) universal value in that limit, hence *κ*_0_(0) might approach a universal value, somewhat above unity. 

## 5. Modeling the Anti-Symmetric Sector

### 5.1. The Super-Ensemble

The (two-degree of freedom) *κ*-distribution may also be understood as follows. Consider two Gaussian uncorrelated degrees of freedom in a thermal bath of fluctuating statistical temperature *β*^–1^. Their PDF is

(30) $$\frac{\beta }{\pi }\exp\left[-\beta (u_{R}^{2}+u_{L}^{2})\right]$$

Following [15,17], we may imagine that statistical temperature *β*^–1^ is a random variable, over a super-ensemble of thermal baths, with probability density *p_θ_*(*β*):

(31) $$p_{\theta }(\beta )=\frac{{\left(\kappa_{0}\theta^{2}\right)}^{\kappa_{0}+1}}{\Gamma (\kappa_{0}+1)}\beta^{\kappa_{0}}\exp[-\kappa_{0}\theta^{2}\beta ]$$

The *κ*-distribution given in Equation (23) follows from averaging (30) over the super-ensemble of random statistical temperature following (31).

The super-ensemble with PDF given by Equation (31) has the following properties. 

(32) $$\langle \beta^{-1}\rangle =\theta^{2},\;\mathrm{for}\;\kappa_{0}>0$$

Secondly, the standard deviation of the random statistical temperature *β*^–1^ is given by

(33) $$\sigma^{2}(\beta^{-1})=\frac{\kappa_{0}}{\kappa_{0}-1}\theta^{4},\;\mathrm{for}\;\kappa_{0}>1$$

In what follows we shall need only *κ*_0_ > 1.

### 5.2. A Minimal Model for the ao Component

The starting point is the condition (22) on the *ao* component of the joint probability distribution for *u_R_* and *u_L_*, stemming from the homogeneity of turbulence. Hence, we are looking for a function which satisfies (22) and also respects the symmetries of the *ao* component,Equations (12) and (13).

Consider two Gaussian uncorrelated degrees of freedom of statistical temperature *β*^–1^. Their PDF is given by Equation (30) which we repeat here:

(34) $$\frac{\beta }{\pi }\exp\left[-\beta (u_{R}^{2}+u_{L}^{2})\right]$$

We want to build a solution of (22) and (12), (13) for the *ao* component, out of (34) in a simple way. The function

(35) $$\frac{\beta }{\pi }\exp\left[-\beta (u_{R}^{2}+u_{L}^{2})\right]\beta^{1/2}(u_{R}-u_{L})$$

satisfies Equations (12) and (13) for the *ao* component, that is, it is consistent with the symmetries of the *ao* component. However, it does not satisfy (22). Next, we may add a factor which is invariant under (12) and (13) for the *ao* component. We do so in the form of a series expansion of the invariant variable *βu_R_u_L_* (that is, invariant under a simultaneous change of sign and an exchange of the velocity variables),

(36) $$\begin{array}{l} \frac{\beta }{\pi }\exp\left[-\beta (u_{R}^{2}+u_{L}^{2})\right]\beta^{1/2}(u_{R}-u_{L})\times \\ \;\times C_{ao}(1+a_{1}\beta u_{R}u_{L}+a_{2}\beta^{2}u_{R}^{2}u_{L}^{2}+a_{3}\beta^{3}u_{R}^{3}u_{L}^{3}+a_{4}\beta^{4}u_{R}^{4}u_{L}^{4}+a_{5}\beta^{5}u_{R}^{5}u_{L}^{5}+\ldots ) \end{array}$$

One may verify that only the product of variables, *βu_R_u_L_*, may be involved in order to satisfy (22), no invariant combinations such *u_R_*^2^+*u_L_*^2^ or *u_R_*^4^+*u_L_*^4^ can be introduced, at least order by order. One then finds that the function

(37) $$\begin{array}{l} \frac{\beta }{\pi }\exp\left[-\beta (u_{R}^{2}+u_{L}^{2})\right]\beta^{1/2}(u_{R}-u_{L})\times \\ \;\times C_{ao}(1+2\beta u_{R}u_{L}+a_{2}[\beta^{2}u_{R}^{2}u_{L}^{2}+\frac{2}{3}\beta^{3}u_{R}^{3}u_{L}^{3}]+a_{4}[\beta^{4}u_{R}^{4}u_{L}^{4}+\frac{2}{5}\beta^{5}u_{R}^{5}u_{L}^{5}]+\ldots ) \end{array}$$

satisfies (22), for any *C_ao_*, *a*_2_, *a*_4_, etc. Hence, Equation (37) is a candidate for the *ao* component model. The minimal choice is *a*_2_=*a*_4_=0, although the more general models (37) may be perfectly interesting and these constants can be fixed through imposing suitable asymptotic behavior. In all, we may proceed with the function

(38) $$C_{ao}\frac{\beta }{\pi }\exp\left[-\beta (u_{R}^{2}+u_{L}^{2})\right]\beta^{1/2}(u_{R}-u_{L})\times (1+2\beta u_{R}u_{L})$$

which satisfies (22) and (12), (13) for the *ao* component and contains no free new parameters. The normalizing factor *C_ao_* will be fixed shortly in terms of the skewness *S* of the velocity derivative distribution.

Now, a superposition of functions (37) will also satisfy (22) and (12), (13) for the *ao* component, as they are linear conditions. We may now imagine that the statistical temperature *β*^–1^ is a random variable with probability density *p_θ_*(*β*) given by (31), that is, the super-ensemble that leads to the kappa-distribution in the symmetric case. Hence we have

(39) $$p_{ao}(u_{R},u_{L})=C_{ao}\times \int_{0}^{\infty }d\beta p_{\theta }(\beta )\frac{\beta^{3/2}}{\pi }(u_{R}-u_{L})(1+2\beta u_{R}u_{L})\exp[-\beta (u_{R}^{2}+u_{L}^{2})]$$

The result is

(40) $$\begin{array}{l} p_{ao}(u_{R},u_{L})=C_{ao}\times \frac{\Gamma (\kappa_{0}+\frac{5}{2})}{\pi \kappa_{0}^{2}\theta^{2}\Gamma (\kappa_{0})}\times \\ \;\times \frac{u_{R}-u_{L}}{\kappa_{0}\theta^{2}}\left(1+\frac{u_{R}^{2}+(2\kappa_{0}+5)u_{R}u_{L}+u_{L}^{2}}{\kappa_{0}\theta^{2}}\right){\left(1+\frac{u_{R}^{2}+u_{L}^{2}}{\kappa_{0}\theta^{2}}\right)}^{-\kappa_{0}-7/2} \end{array}$$

This is a model of the *ao* component that is built by the super-ensemble distribution of statistical temperatures (31) from a minimal model Gaussian-type of model that respects the conditions (12), (13) for the *ao* component and (22).

As mentioned above the constant *C_ao_* may only be fixed through information from the structure functions. Let us then calculate the third order structure functions by Equation (16):

(41) $$B_{3}=\iint {\left(u_{R}-u_{L}\right)}^{3}p_{ao}(u_{R},u_{L})du_{R}du_{L}$$

**Figure 3:**
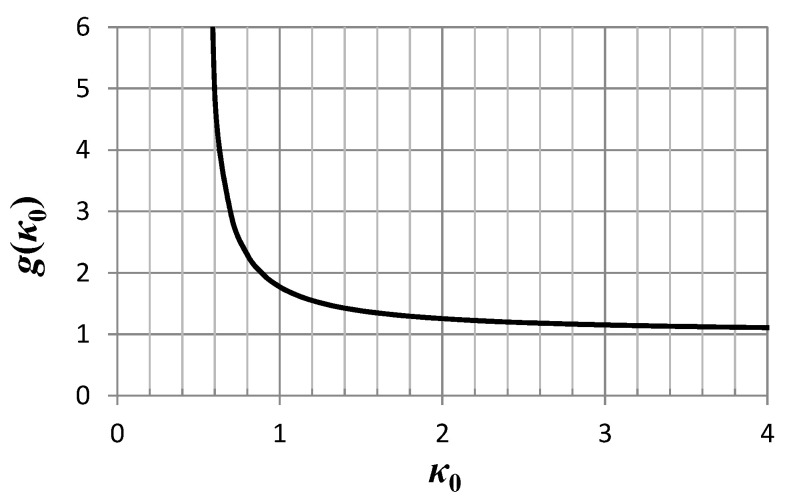
Plot of the function *g*(*κ*_0_) defined by Equation (43). *g* becomes infinite as *κ*_0_ approaches the value 0.5.

One finds

(42) $$B_{3}=-3C_{ao}g(\kappa_{0})\theta^{3}$$

where we have defined the function

(43) $$g(\kappa_{0})=\frac{\kappa_{0}^{1/2}\Gamma (\kappa_{0}-\frac{1}{2})}{\Gamma (\kappa_{0})}$$

which is a characteristic of the model, that is, model (38) in our case. This function is plotted in Figure 3. For *κ*_0_→∞, *g*(*κ*_0_)→1 while for *κ*_0_→1/2, *g*(*κ*_0_)→∞. We note that it need only be that *κ*_0_ > 1/2. 

One should recall that from the symmetric sector we learned that *B*_2_ = *θ*^2^ (Equation (24)). Then by Equation (5) we have that

(44) $$C_{ao}=-\frac{S}{3g(\kappa_{0}(0))}$$

where *κ*_0_(0) is fixed in terms of the Reynolds number through Equation (29). This completes the construction/modeling of the *ao* component of the joint probability distribution of *u_R_* and *u_L_*. One may note that, similarly to the flatness factor, the skewness *S* is a function of the Reynolds number: *S* = *S*(Re_λ_). For example, a form of power law dependence in terms of the Reynolds number is usually assumed, see e.g., [5] and the references there in. The authors in [5] estimate that –*S* = 0.32(Re_λ_)^0.11^. More recently, it has been suggested that the skewness factor actually converges to a universal value for large Reynolds numbers, see e.g., [21]. Whatever the dependence of the skewness and flatness factor on Re_λ_ might be, that dependence in inherited by the two points PDF of the velocity field.

We may finally write down the complete two-point PDF of the velocity field, according to our proposals, following from Equations (15), (23) and (40) and (43), (44):

(45) $$\begin{array}{l} p(u_{R},u_{L})=\frac{\kappa_{0}+1}{\pi \kappa_{0}\theta^{2}}{\left(1+\frac{u_{R}^{2}+u_{L}^{2}}{\kappa_{0}\theta^{2}}\right)}^{-\kappa_{0}-2}-S\frac{\Gamma (\kappa_{0}(0))}{3\kappa_{0}^{1/2}(0)\Gamma (\kappa_{0}(0)-\frac{1}{2})}\times \frac{\Gamma (\kappa_{0}+\frac{5}{2})}{\pi \kappa_{0}^{2}\theta^{2}\Gamma (\kappa_{0})}\times \\ \;\times \frac{u_{R}-u_{L}}{\kappa_{0}\theta^{2}}\left(1+\frac{u_{R}^{2}+(2\kappa_{0}+5)u_{R}u_{L}+u_{L}^{2}}{\kappa_{0}\theta^{2}}\right){\left(1+\frac{u_{R}^{2}+u_{L}^{2}}{\kappa_{0}\theta^{2}}\right)}^{-\kappa_{0}-7/2} \end{array}$$

### 5.3. The General Validity of the Function g(κ_0_) of Equation (43)

Consider building the *ao* component through the super-ensemble via a density function associated with a statistical temperature *β*^–1^ (according to the inverse temperature PDF *p_θ_*(*β*) given in Equation (31)). Now, if no dimensionful scales exist other than *β*^–1^, that density function must be a function of the variables *β*^1/2^*u_R_*, *β*^1/2^*u_L_*. That is,

(46) $$p_{ao}(u_{R},u_{L})=\int_{0}^{\infty }d\beta p_{\theta }(\beta )\beta \rho (\beta^{1/2}u_{R},\beta^{1/2}u_{L})$$

for some density *ρ*, that must be such that Equation (47) obeys the conditions (12), (13) for the *ao* component and (22). 

Consider now calculating *B*_3_ for the function (46) via (41). 

(47) $$B_{3}=\int_{0}^{\infty }d\beta p_{\theta }(\beta )\times \iint \rho (\beta^{1/2}u_{R},\beta^{1/2}u_{L}){\left(u_{R}-u_{L}\right)}^{3}\beta du_{R}du_{L}\;$$

Changing variables according to *u_R_*→*β*^–1/2^*u_R_*, *u_L_*→*β*^–1/2^*u_L_* we have

(48) $$B_{3}=\int_{0}^{\infty }d\beta p_{\theta }(\beta )\times \beta^{-3/2}\times \iint \rho (u_{R},u_{L}){\left(u_{R}-u_{L}\right)}^{3}du_{R}du_{L}$$

that is,

(49) $$B_{3}=C_{\rho }\times \langle \beta^{-3/2}\rangle =C_{\rho }\times g(\kappa_{0})\;\theta^{3},\;C_{\rho }=\iint \rho (u_{R},u_{L}){\left(u_{R}-u_{L}\right)}^{3}du_{R}du_{L}$$

where *g*(*κ*_0_) is precisely the function given by Equation (43), and *C_ρ_* is a constant associated with density *ρ*. We note that *g*(*κ*_0_) is essentially given by the mean value of *β*^–3/2^ through the super-ensemble PDF given by Equation (31). In all, the function *g*(*κ*_0_) follows only from the specific PDF for *β* of the super-ensemble, given by (31), and that the only dimensionful parameter is *θ*, or equivalently *β*.

### 5.4. The Third Order Structure Function B_3_(r) and κ_0_(r); κ_0_(r) and Closures of Turbulence

Equations (42) and (44) imply that

(50) $$B_{3}=S\frac{g(\kappa_{0})}{g(\kappa_{0}(0))}\theta^{3}$$

One should bear in mind that both *θ* and *κ*_0_ depend on the distance *r.* Recalling Equation (24) we may write (49) in the following forms

(51) $$B_{3}(r)=S\frac{g(\kappa_{0}(r))}{g(\kappa_{0}(0))}{\left(B_{2}(r)\right)}^{3/2}$$

(52) $$g(\kappa_{0}(r))=\frac{B_{3}(r)}{S\;{\left(B_{2}(r)\right)}^{3/2}}g(\kappa_{0}(0))$$

A few comments are in order.

**Comment** **1.** *Via Equation (52), κ_0_(r) is defined in terms of the functions B_3_ and B_2_ along with quantities that depend only on the Reynolds number. Therefore, κ_0_(r) depends on the additional information about turbulence that is stored in the third order structure function B_3_*.

**Comment** **2.** *As we showed in the previous section the function g given by (43) holds for any model that derives from the super-ensemble with the PDF (31), as long as there is only one dimensionful parameter. On the other hand, a super-ensemble with a PDF different than (31) but still involving a single temperature parameter, will still lead to Equations (51) and (52) but a different function g*.

**Comment** **3.** *From Equations (3) and (7) we see that the ratio B_3_(r)/(B_2_(r))^3/2^ is constant in the inertial subrange, the same as it is in the small distances. Hence, κ_0_(r) must be constant through the inertial subrange scales.*

**Figure 4:**
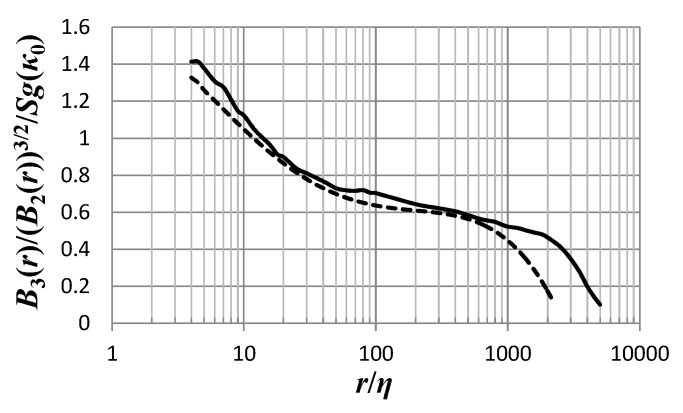
The ratio *g*(*κ*_0_(0))*B*_3_(*r*)/(*B*_2_(*r*))^3/2^/*S* as it follows from DNS data [6] for Reynolds number Re*_λ_*= 1131 (continuous line) and Re*_λ_*= 732 (dashed line), and Equations (43) and (29).

**Comment** **4.** *As far as phenomenology is concerned, knowing B_3_ and B_2_ for all r means knowing the whole of the function κ_0_(r). Now, modern DNS data [5,6,7,8,9] allow us to calculate the ratio B_3_(r)/(B_2_(r))^3/2^ at decently high Reynolds numbers so that the K41 Equations (3) and (7) are approximately realized. Indeed, through the data of [6] we calculate the r.h.s. of Equation (52), for Reynolds numbers Re_λ_ = 732 and Re_λ_ = 1131, the two highest Reynolds numbers for the resolution kη~1, where the length η = (ν^3^/ε)^1/4^ is the Kolmogorov dissipation range length scale, i.e., the scale of the smallest eddies in the flow. The result is shown in Figure 4 as a function of r/η.*

*The result is that for r larger than ~15η, the r.h.s. of (52) becomes smaller than 1, for the case of the Reynolds number Re_λ_ = 1131, while the same happens for r larger than ~11η, for the case of the Reynolds number Re_λ_ = 732. That means that those values cannot be described by the function g(κ_0_) of Equation (43), which is always greater than 1. Hence, we conclude that the minimal model (38), or essentially any model constructed out of the super-ensemble with PDF given by (31), may describe turbulence only for distance r at most ~10η, depending on the Reynolds number. We may also say that the model acquires a greater regime of applicability as we look at cases of higher Reynolds numbers. Those distances essentially cover the dissipation subrange of isotropic turbulence. That means that the minimal model (36), and any model that rests on the PDF (31), is a dissipation subrange model.*

**Figure 5:**
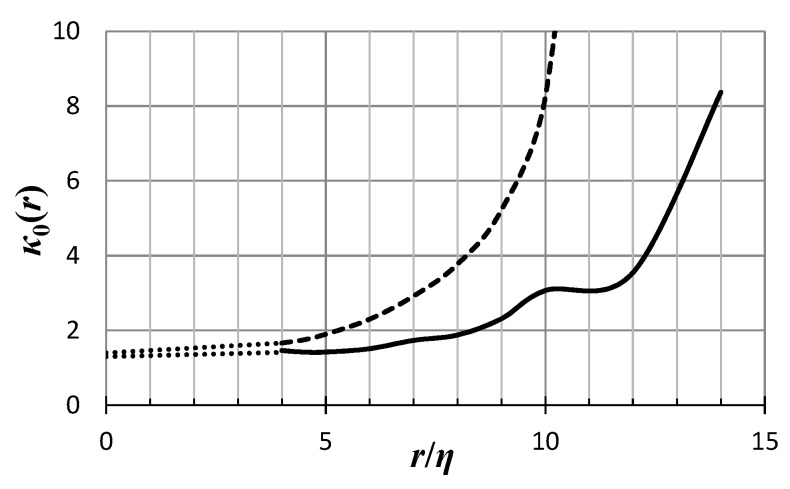
The kappa-index *κ*_0_(*r*) as a function of *r*/*η* derived from DNS data [6] for Reynolds number Re*_λ_*= 1131 (continuous line) and Re*_λ_*= 732 (dashed line), and Equation (43). The kappa-index starts off with an estimated value *κ*_0_(0) ~ 1.4 and *κ*_0_(0) ~ 1.66, respectively, and increases without bound. The dotted lines indicate the expected behavior in the region where we do not have information from the DNS, through interpolation.

*The function κ_0_(r) corresponding to the results shown in Figure 4 is shown in Figure 5 as a function of r/η, for the Reynolds number Re_λ_ = 1131 (continuous line) and Re_λ_ = 732 (dashed line). The data of [6] allow calculations only for r/η ≥ 4. The expected behavior of κ_0_(r) for smaller distances, on the basis of our previous discussion, is given by a dotted line. On the other end, as the r.h.s. of (52) approaches the value 1 the kappa-index κ_0_ becomes infinitely large.*

**Comment** **5.** *A pre-defined function κ_0_(r), e.g., according to some independent assumption, amounts to what is known in the turbulence literature as a ‘closure scheme’ [2] via (51). At the level of structure functions, the statistical theory of isotropic turbulence amounts to an infinite set (a hierarchy) of coupled dynamical equations: Due to the non-linear term in the Navier–Stokes equation the dynamical equation for the function B_n_(r) involves the function B_n+1_(r). A relation, such as (51), that gives the higher order structure function B_3_ in terms of the lower order structure function B_2_, ‘closes off’ the infinite hierarchy at the first non-trivial level, the so-called Karman–Howarth equation [1,2,10]. Then, B_2_(r) can be determined. Given that a closure is, at best, a claim for an approximate solution of isotropic turbulence, knowing or deriving κ_0_(r) from some sort of first principles is a highly non-trivial ambition.*

**Comment** **6.** *Very good closures (for example, they encapsulate the bottleneck effect [9]) do exist in wave-number space, the so-called EDQNM (eddy-damped, quasi-normal markovian) type of closures [22], stemming from Kraichnan’s direct interaction approximation (DIA) [23]. The construction of κ_0_(r) through the EDQNM, via Equation (52), is the nearest one may come to a derivation from first principles. The related analysis is left for future work.*

**Comment** **7.** *The Karman–Howarth equation [2,10] reads*

(53) $$\frac{\partial B_{2}}{\partial t}=\frac{1}{r^{4}}\frac{\partial }{\partial r}\left[r^{4}\left(2\nu \frac{\partial B_{2}}{\partial r}-\frac{1}{3}B_{3}\right)\right]-\frac{4}{3}\varepsilon +\frac{4}{3}\varepsilon_{\mathrm{forcing}}(r)$$

*where ε_forcing_(r) is a rate at which energy (per unit mass) is fed into the system at scale r. Usually the forcing term is negligible in the inertial range and the smaller scales. Assuming that a stationary state had been reached, due to the forcing which balances the dissipation of energy via the viscous forces, Equation (53) can be integrated to the form*

(54) $$6\nu \frac{\partial B_{2}}{\partial r}-B_{3}-\frac{4}{5}\varepsilon r+\frac{4}{3}r^{-4}\int_{0}^{r}dr^{\prime }{r^{\prime }}^{4}\varepsilon_{\mathrm{forcing}}(r^{\prime })=0.$$

*Using now Equations (24) and (51), we have*

(55) $$6\nu \frac{\partial \theta^{2}(r)}{\partial r}-\frac{g(\kappa_{0}(r))}{g(\kappa_{0}(0)}\theta^{3}(r)-\frac{4}{5}\varepsilon r+4r^{-4}\int_{0}^{r}dr^{\prime }{r^{\prime }}^{4}\varepsilon_{\mathrm{forcing}}(r^{\prime })=0.$$

*That is, the fundamental Karman–Howarth equation translates to a differential equation relating θ^2^(r) and κ_0_(r). A dynamical version of Equation (55) follows from Equation (53) and Equations (24) and (51).*

**Comment** **8.** *Equation (55) makes clear that the long distance behavior of the structure function B_3_(r), and hence of κ_0_(r), is dependent on the energy feeding mechanism of large scales. Although, the usual large-scale forcing mechanism, see e.g., [6], produces a negligible ε_forcing_(r) in the small scales, its integral in Equation (55) is not negligible and is also dependent on the forcing scheme. An independent source of information for κ_0_(∞) comes for the single-point PDF of the velocity field and is discussed below*.

### 5.5. Deriving a Model of the Distribution of ∂u_x_/∂x

A highly important quantity in turbulence is the probability distribution of the (longitudinal) velocity derivative, ∂*u_x_*/∂*x*. As it is clear from the complete two-point PDF in Equation (45) equations the velocity variables are scaled by *θ*√*κ*_0_. In order to derive the PDF for ∂*u_x_*/∂*x* one may change variables to

(56) $$U_{+}=\frac{u_{R}+u_{L}}{\theta \sqrt{\kappa_{0}}},\;U_{-}=\frac{u_{R}-u_{L}}{\theta \sqrt{\kappa_{0}}}$$

and integrate over all values of *U_+_*, using the fact that

(57) $$du_{R}du_{L}=\frac{1}{2}\kappa_{0}\theta^{2}dU_{+}dU_{-}.$$

Now, we have that *θ*^2^ = *B*_2_ (Equation (24)). Clearly, we are interested in small distances, hence we need to use the small distance form of the function *B*_2_, given by Equation (2). Indeed, by (2) and (1) we have that

(58) $$U_{-}=\frac{\sqrt{15\nu }}{\sqrt{\kappa_{0}(0)}\sqrt{\varepsilon }}\frac{\partial u_{x}}{\partial x}=\frac{1}{\sqrt{\kappa_{0}(0)}\sigma }\frac{\partial u_{x}}{\partial x}$$

where *σ* is the standard deviation of ∂*u_x_*/∂*x*. (Here, the small distances kappa index *κ*_0_(0) is involved.) Indeed, by (2), we have that

(59) $$\sigma^{2}=\langle {\left(\frac{\partial u_{x}}{\partial x}\right)}^{2}\rangle =\frac{\varepsilon }{15\nu },$$

that is, *U*_–_ is essentially the normalized derivative of the velocity divided by √*κ*_0_(0). It is important to note that the expectation value of the derivative of the velocity is zero by the homogeneity of turbulence. In the present context this is realized as each of the following integrals vanishes:

(60) $$\iint \left(u_{R}-u_{L})p_{se}(u_{R},u_{L}\right)du_{R}du_{L}+\iint \left(u_{R}-u_{L})p_{ao}(u_{R},u_{L}\right)du_{R}du_{L}$$

The first integral vanishes by the evenness of the *se* function, while the second integral vanishes by condition (22) on the *ao* function. Integrating over *U*_+_ according to measure (57) we have that the PDF for ∂*u_x_*/∂*x* in terms of the *U*_–_ variable is

(61) $$\begin{array}{l} p_{U_{-}}(U_{-})=\frac{\Gamma (\kappa_{0}(0)+\frac{3}{2})}{\sqrt{2\pi }\kappa_{0}(0)\Gamma (\kappa_{0}(0))}{\left(1+\frac{1}{2}U_{-}^{2}\right)}^{-\kappa_{0}(0)-3/2}\\ \;-S\frac{\left(\kappa_{0}(0)+1)\Gamma (\kappa_{0}(0)\right)}{12\sqrt{2\pi \kappa_{0}(0)}\Gamma (\kappa_{0}(0)-\frac{1}{2})}U_{-}^{}[6-(2\kappa_{0}(0)+1)U_{-}^{2}]{\left(1+\frac{1}{2}U_{-}^{2}\right)}^{-\kappa_{0}(0)-3} \end{array}$$

One may verify that (60) is everywhere positive and satisfies the conditions

(62) $$\begin{array}{l} \int p_{U_{-}}(U_{-})dU_{-}=1,\;\int p_{U_{-}}(U_{-})U_{-}dU_{-}=0,\\ \int p_{U_{-}}(U_{-}){\left(\sqrt{\kappa_{0}(0)}U_{-}\right)}^{2}dy=1,\;\int p_{U_{-}}(U_{-}){\left(\sqrt{\kappa_{0}(0)}U_{-}\right)}^{3}dU_{-}=S \end{array}$$

which is what one would expect from a PDF for ∂*u_x_*/∂*x*, given definition (57). *κ*_0_(0) is given by Equation (29) as a function of the Reynolds number.

The PDF for *U*_–_ given by (61) translates into the PDF for the (normalized) velocity derivative by

(63) $$p_{\partial u/\partial x/\sigma }\left(\frac{\partial u_{x}}{\sigma \;\partial x}\right)=\frac{1}{\sqrt{\kappa_{0}(0)}}p_{U_{-}}\left(\frac{\frac{\partial u_{x}}{\sigma \;\partial x}}{\sqrt{\kappa_{0}(0)}}\right)$$

where *σ* is defined by (59). Using the DNS data of [5] we compare the predictions for the PDF of the velocity derivative from the present model. The equations needed are: (61) and (63) and formula (29) for the small scale kappa-index *κ*_0_(0). We use the data for Re*_λ_* = 675, which is the highest Reynolds number with resolution *k*_max_*η* ~ 2, while higher Reynolds number runs were done with *k*_max_*η* ~ 1. In particular, we set –*S* = 0.64 [5]. The higher resolution is important for the specific purpose of finding the PDF of the velocity derivative because the curve changes rather significantly as the resolution increases [5].

**Figure 6:**
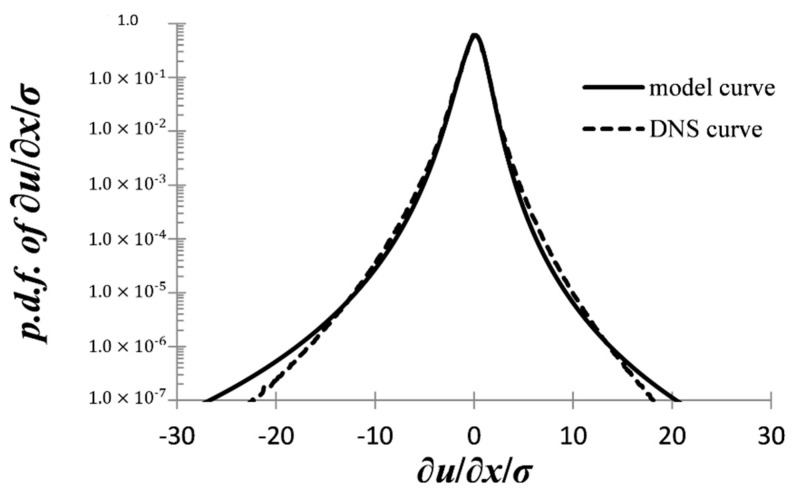
Continuous line: The model probability density function (PDF) of the normalized (longitudinal) velocity derivative for Re*_λ_* = 675, as it follows from Equations (61), (63), (29). Dashed line: The corresponding DNS data for Re*_λ_* = 675 [5].

The results are shown in Figure 6, where the continuous line shows the curve of the present model, Equations (61), (63), and (29), while the dashed line is the DNS curve. The model curve is rather acceptable ∂*u_x_*/∂*x*/*σ* roughly between ±10, where the PDF is larger than 10^–5^, but deviates in the tails of the distribution. The model curve appears very good for the interval ∂*u_x_*/∂*x*/*σ* roughly between ±2. One should note that the effect of the increasing resolution is to bring the DNS curve closer to the model curve. Overall, one may say that the whole construction works impressively well: There is nothing in our set up containing a priori the behavior of the DNS curve in Figure 6, while even the value of constant *κ*_0_(0) was self-consistently calculated from flatness DNS data and not just fiddled with for best fit. Due to the minimal nature of the model (38), we shall not proceed to further comparing the model curves with DNS results for different Reynolds numbers, as the more general family of models given by Equations (37) do produce different curves for the PDF of the velocity derivative, and their effectiveness will be explored in a separate publication.

## 6. The PDF of *u_x_*

According to the discussion of Section 3, the PDF for the velocity *u_x_* at any given point is given by the integration of the joint PDF for the velocity of two points given by Equation (45). The result is

(64) $$p(u)=\frac{\Gamma (\kappa_{0}+\frac{3}{2})}{\sqrt{\kappa_{0}\theta^{2}}\sqrt{\pi }\kappa_{0}\Gamma (\kappa_{0})}{\left(1+\frac{u^{2}}{\kappa_{0}\theta^{2}}\right)}^{-\kappa_{0}-3/2}\;,$$

which is nothing but the kappa-distribution PDF for one degree of freedom. This makes sense, as the *ao* component in the two-point PDF does not contribute to result for the one-point PDF by condition (24). 

One should bear in mind that both the statistical temperature and the kappa-index in (64) still carry the dependence on *r*, as the derivation at this stage was done within the ensemble of eddies of sizes up to *O*(*r*). That may be phenomenologically useful as in actual physical flows, e.g., space plasma, information may actually be available from a region of size *r* not containing the entire flow.

On the other hand, looking to answer—within our setup—the question about the one-point PDF of the velocity field in the ideal isotropic flow, it makes more sense to include the whole of the flow, that is, let *r*→∞. ‘Infinite distance’ essentially means adequately larger than the integral scale of turbulence [10], which is the length scale of the correlation of the velocity field. Then, according to Equations (5) and (24), we have

(65) $$\theta^{2}=2{u^{\prime }}^{2}.$$

That is, we have

(66) $$p(u)du=\frac{\Gamma (\kappa_{0}(\infty )+\frac{3}{2})}{\sqrt{2\pi }{\left[\kappa_{0}(\infty )\right]}^{3/2}\Gamma (\kappa_{0}(\infty ))}{\left(1+\frac{u^{2}}{2\kappa_{0}(\infty ){u^{\prime }}^{2}}\right)}^{-\kappa_{0}(\infty )-3/2}d\left(\frac{u}{u^{\prime }}\right)\;$$

where *κ*_0_(∞) is the infinite distance value of the index *κ*_0_. Note that that *u’* is the standard deviation of *u_x_* (Equation (4)). Equation (66) is the PDF of the normalized velocity, i.e., of unit standard deviation. 

It is worth to note the following. We could have started with the one-degree of freedom PDF (64) and looked for a value for *θ*^2^ knowing nothing about the previous two-point PDF analysis. The mean value of *u_x_* according to (64) is, of course, zero, while the mean value of *u_x_*^2^ is ½ *θ*^2^. However, if (64) is to describe the PDF of the velocity at a point within an isotropic turbulent flow with r.m.s. value of the velocity equal to *u’*, or equivalently with energy per unit mass equal to (3/2)*u’*^2^, then we should identify ½ *θ*^2^ with *u’*^2^ and (64) necessarily follows. Hence, the result (64) is verified independently, within our framework.

It is now straightforward to show that the flatness of the *u_x_*-distribution is given by

(67) $$F_{u}=\frac{3\kappa_{0}(\infty )}{\kappa_{0}(\infty )-1},$$

analogously to equation (27). That is,

(68) $$\kappa_{0}(\infty )=\frac{F_{u}}{F_{u}-3}.$$

DNS data [5] implies that *F_u_* is below but near and below 3, i.e., nearly but not actually Gaussian. That means that *κ*_0_(∞) is large and negative and large: the data of [5] imply *κ*_0_(∞) between −16 and −74. 

Now in the previous section we found that that *κ*_0_(*r*) starts with positive values in the small distances and increases, although due to restrictions of the minimal model (36) we could not ‘see’ beyond *r*~10*η*. Nonetheless, for *κ*_0_(*r*) to become negative it is necessary for either *κ*_0_(*r*) to become discontinuous, or to pass through the value zero. The first case introduces a bigger unknown in the problem, regarding the nature and the size of the discontinuity, while the second case is merely physically unacceptable. Given that (64) becomes Gaussian for both infinite limits *κ*_0_(*r*)→±∞, and the DNS data estimates given in the previous paragraph, one concludes that the present construction essentially predicts that *κ*_0_(∞)=+∞,(∞)=+∞, as the nearest approximation to the results of the DNS explained in the previous paragraph. That is, the single-point PDF of the velocity field is Gaussian. 

## 7. Summary and Discussion

In the present work, we construct a model for the two-point PDF of the velocity field for the ideal isotropic flow, based on the theory of the kappa distribution and the concept of superstatistics [15]. Due to the length of the work, a summary of main points and result is given in the form of the Table 1 below.

The main conclusions of this paper are:

1. The two-point PDF of the velocity field is a sum of a symmetric-even and an antisymmetric-odd part, as a result of reflection symmetry. The antisymmetric part obeys a non-trivial integral condition, as a result of the homogeneity of the flow.
2. The symmetric part, which gives rise to the even order structure functions, may be modeled by a kappa distribution of two degrees of freedom (DOF). The temperature parameter is given by the second order structure function. That is, the ensemble of eddies with size up to *O*(*r*) has a statistical temperature *B*_2_(*r*) and an *r*-dependence kappa index.
3. Using data from the DNS of isotropic turbulence and the calculated flatness factor of the velocity gradient distribution from the model, one deduces the dependence of the kappa index of the smallest eddies ensemble on the Reynolds number.
4. The antisymmetric part, which gives rise to the odd order structure functions, may be modeled by a minimal model constructed on the basis of symmetry properties of the antisymmetric-odd part and the non-trivial integral condition mentioned in Conclusion 2, and utilizing the superstatistics construction. The ensemble of eddies with size up to *O*(*r*) has an *r*-dependence kappa index that derives from third order structure function.
5. The fundamental Karman-Howarth equation of isotropic turbulence translates to a differential relation between the *r*-dependent statistical temperature and kappa-index.
6. The relation between *B*_2_(*r*), *B*_3_(*r*) and the *r-*dependent kappa index is not peculiar to the minimal model: Any model deriving from the superstatistics construction, in particular the (gamma) probability distribution of the inverse statistical temperature, results in the same relation.
7. By the specific form of the relation between *B*_2_(*r*), *B*_3_(*r*) and the *r-*dependent kappa index, the DNS data imply that the constructed model is a dissipation subrange model, not applicable e.g., in the inertial subrange, although the range of validity increases with the Reynolds number. By Conclusion 5, the only way out is the modification of the probability distribution of the inverse statistical temperature in the super-ensemble.
8. The two-point PDF of the velocity field allows the derivation of the velocity gradient PDF In the context of the minimal model, the resulting PDF agrees well within six orders or magnitude with the numerical velocity gradient PDF from the DNS.
9. The one-point PDF of the velocity field is a kappa distribution when defined in a finite volume, in particular, of the order of magnitude of the integral scale of turbulence, or smaller. In the ideal isotropic case of infinite volume, the most plausible deduction from the present construction is that the one-point PDF is Gaussian.

As it has been already noted, this work is only the first step in completing an effective model for the PDFs of the velocity field of isotropic turbulence, on the basis of kappa distribution and the superstatistics construction. First of all, models more complicated than the minimal model, already given in this work, need to be investigated with respect to improving further the agreement between the predicted PDF of the velocity gradient and the DNS. Secondly, on a more basic level, one needs to deal with the fact that the derived PDFs are models valid only within the dissipative subrange, due to the specific (gamma) distribution of the inverse statistical temperature in the superstatisics construction. Hence, other inverse statistical temperature distributions need to be investigated. Finally, an obvious deficiency of the proposed PDFs is that they have only very low order moments of the velocity and the velocity increment between two points, for the values of the kappa index consistent with DNS data, as the associated integrals diverge for the general moment. That cannot be correct in the context of turbulence: Arbitrarily high velocities need to be exponentially cut off, as they are associated with arbitrarily small eddies, which do not exist. That calls for a modification of the inverse statistical temperature distribution, which could simultaneously improve the agreement with the DNS data for the velocity gradient distribution, without destroying the kappa distribution-type of behavior of the PDFs for smaller values of the velocities.

## Figures and Tables

**Table 1:** A summary of main points and results of the paper.

**Two-point PDF for velocity field (follows from invariance under reflections)** $p(u_{R},u_{L})=p_{se}(u_{R},u_{L})+p_{ao}(u_{R},u_{L})$		
Component	$p_{se}(u_{R},u_{L})$	$p_{ao}(u_{R},u_{L})$
Symmetry	$\begin{array}{l} p_{se}(u_{R},u_{L})=+p_{se}(u_{L},u_{R})\\ p_{se}(u_{R},u_{L})=+p_{se}(-u_{R},-u_{L}) \end{array}$	$\begin{array}{l} p_{ao}(u_{R},u_{L})=-p_{ao}(u_{L},u_{R})\\ p_{ao}(u_{R},u_{L})=-p_{ao}(-u_{R},-u_{L}) \end{array}$
Structure function	Even order, e.g., *B*_2_(*r*)	Odd order, e.g., *B*_3_(*r*)
Integral condition	$\iint p_{se}(u_{R},u_{L})du_{R}du_{L}=1$	$\int p_{ao}(u,v)dv=0,\;\mathrm{for}\;\mathrm{all}\;u$
Modeled by	Two DOF *κ*-distribution	Novel function, the minimal model, given by Equation (38), built out of symmetry conditions, simplicity and the super-ensemble.
Intensive variable translation in turbulence	Temperature parameter: *θ*^2^ = *B*_2_(*r*), Equation (24)	*κ*-index: *κ*_0_(*r*), Equation (51): $g(\kappa_{0}(r))=\frac{B_{3}(r)}{S\;{\left(B_{2}(r)\right)}^{3/2}}g(\kappa_{0}(0))$ The characteristic function of the model, *g*(*κ*_0_): $g(\kappa_{0})=\frac{\langle \beta^{-3/2}\rangle }{\theta^{3}}=\frac{\kappa_{0}^{1/2}\Gamma (\kappa_{0}-\frac{1}{2})}{\Gamma (\kappa_{0})}$ This function obeys *g*(*κ*_0_) ≥ 1, which imposes restrictions on the admissible values of the ratio *B*_3_(*r*)/(*B*_2_(*r*))^3/2^ in the context of the model. The function *g* holds for any model that derives from the super-ensemble with a PDF given by Equation (31). For super-ensemble PDFs different than Equation (31), the function *g* will be different but Equations (51) and (52) will still hold.
**Physical description: The ensemble of eddies of size up to *O*(*r*), has a statistical temperature *θ*^2^(*r*) and kappa-index *κ*_0_(*r*) associated with the second and third order structure functions *B*_2_(*r*) and *B*_3_(*r*).**		
Moments of the ∂*u_x_*/∂*x* distribution	Flatness *F* = *F*(Re*_λ_*)	Skewness *S* = *S*(Re*_λ_*)
The moments imply:	The small distance (*r* = 0) kappa-index is fixed from direct numerical simulations (DNS) of isotropic turbulence as a function of the Reynolds number: *κ*_0_(0) = *κ*_0_(0)(Re*_λ_*).	
The intensive variable translation implies:	Through Equations (52) and (43), DNS data allow the determination of the function *κ*_0_(*r*). The minimal model of Equation (36) describes the DNS for distances *r* less than ~10*η* i.e., it covers the dissipation range (outside of it the condition *g*(*κ*_0_) ≥ 1 is not satisfied by the DNS data). The minimal model is a dissipation range model. The same applies to any model derived by the super-ensemble with PDF given Equation (31).	
*θ*^2^(*r*) and *κ*_0_(*r*) are fundamentally related	The Karman–Howarth differential equation relates the *B*_2_(*r*) and *B*_3_(*r*) of isotropic turbulence, through the intensive variables translation, Equations (24) and (52), and this translates to a fundamental relation between the statistical temperature and the kappa-index functions.	
The *κ*_0_(*r*) and closure schemes of isotropic turbulence	A relationship between *B*_2_(*r*) and *B*_3_(*r*), independent of the Karman–Howarth equation, is a closure scheme for the hierarchy of isotropic turbulence. Postulating a function *κ*_0_(*r*) amounts to a closure. Inversely, a closure of isotropic turbulence produces a function *κ*_0_(*r*). Examples of very effective closures are the eddy-damped quasi-normal Markovian (EDQNM) models.	
The PDF of ∂*u_x_*/∂*x*	The two-point function and the translation of the intensive variables, allow the determination of the PDF of ∂*u_x_*/∂*x* according to the kappa-distribution and the minimal model. The derived PDF depends on the Reynolds number. It reproduces fairly well the DNS data for this quantity.	
The PDF of *u_x_*	The one-point PDF for the ideal isotropic flow follows from the two-point function, upon integration of one velocity variable for any given *r* = ∞ (the whole of the flow). DNS data imply a near Gaussian distribution (of flatness near but less than 3) which forces *κ*_0_(∞) to be large and negative. Small distances require a positive increasing function *κ*_0_(*r*), making negative values unacceptable. Both *κ*_0_(∞) = ±∞ values are equivalent to Gaussian. *κ*_0_(∞) = +∞, that is, a Gaussian PDF of *u_x_*, is essentially the prediction of the present construction.	
